# The Use of Visual Analogue Scale Score as a Predicting Tool in Differentiating Renal Colic From Lumbar Back Pain

**DOI:** 10.7759/cureus.16377

**Published:** 2021-07-13

**Authors:** Mehmet Caniklioğlu, Muharrem Özkaya

**Affiliations:** 1 Urology, Bozok University Faculty of Medicine, Yozgat, TUR; 2 Urology, Sinop Atatürk State Hospital, Sinop, TUR

**Keywords:** renal colic, low back pain, vas score, differential diagnosis, scoring systems, flank pain

## Abstract

Introduction

Renal colic is often confused with low back pain (LBP) and other pathologies. Computed tomography (CT) is frequently used to reach a definitive diagnosis, but its use increases the exposure to radiation. Researchers have tried to predict urinary stones in patients presenting with flank pain. Several scoring systems have been introduced; however, none of them provide a prediction based on the physical examination of the patient upon initial presentation to the outpatient clinic. In this study, we aimed to investigate whether we can predict the presence of stone with visual analogue scale (VAS) questionnaire during the first admission.

Materials and methods

Patients with complaints of flank pain were started to be followed for three months in our urology clinic. After the definitive diagnosis was made the patients were classified into two groups: renal colic group (group 1; n=36) and the LBP group (group 2; n=30).

Results

In logistic regression analysis, the possibility of renal colic increased 5.4 times more per one-unit increase in the VAS score. In receiver operating characteristic (ROC) analyses, when the VAS was 4.5, the diagnosis of renal colic could be made with 88% sensitivity and 71% specificity.

Conclusion

If the VAS score is ≤ 4 in patients that have flank pain without limitation of movement, it is more likely to manage these patients with a simple medical treatment plan. In these patients, unnecessary ultrasonography (US) scans be reduced by 86.3% and unnecessary CT scans by 88.8%. A VAS score of ≥5 should warn the clinician about the necessity of routine urinary stone examinations.

## Introduction

Urolithiasis is a common disease with a lifetime incidence of 12% and a high cost for healthcare worldwide [1,2]. Although ultrasonography (US) is the first-line diagnostic method in stone diagnosis, it is highly dependent on the attention and experience of the person performing it, and its sensitivity remains low, especially in detecting ureteral and kidney stones [3]. Therefore, non-contrast computed tomography (CT), a gold standard diagnostic method, is frequently used to reach a definitive diagnosis, but its use increases the cost and exposure to radiation [1,4,5].

Renal colic is often confused with low back pain (LBP) and other pathologies [6]. Physical examination and medical history are generally insufficient for diagnosis, and therefore there has been a significant increase in the use of radiological methods, especially CT, in the last decades [7]. Since renal colic is not often a life-threatening pathology, frequent use of CT in its diagnosis is a matter of debate [8]. Some studies have reported that stones were detected in 50% of patients that underwent CT with suspicion of urolithiasis and only 20% of them required intervention [9]. Clinicians have also tried to predict urinary stones in patients presenting with flank pain with the aim of reducing radiation exposure and cost. Some researchers have worked on this issue and tried to introduce some scoring systems [5,9-12]. However, none of these scoring systems provide a prediction based on the physical examination of the patient upon initial presentation to the outpatient clinic. Some physicians can intuit a pre-diagnosis of renal colic based on the verbal information received from the patients, their subjective experiences, and patients’ severity of the pain. Theoretically, renal colic pain is expected to be much more severe than musculoskeletal pain with the exception of lumbar disc herniation pain. At this point, a question of "Is it possible to make an accurate prediction of renal colic based on pain intensity?" comes to mind.

We thought that hypothetically, pain intensity would be higher in patients with renal stones than in patients with LBP and a cutoff value for a visual analogue scale (VAS) score could be found. In this study, we aimed to investigate whether we can predict the presence of stone with a simple and short VAS questionnaire. The main idea of the study was the clinical observation that stone pain is more severe than LBP. We aimed to test the accuracy of this clinical observation.

## Materials and methods

The local ethics committee approved the study (protocol number: 2017-KAEK-189_2019.11.13_14). It was designed as a prospective cohort analysis. Informed consent was obtained from all participants. Patients that applied to our urology outpatient clinic with complaints of flank pain were started to be followed for three months. Except for the VAS scoring of the patients, all examinations and evaluations were performed by a urologist experienced in the follow-up and treatment of urinary stone disease. Prior to the physical examination, the secretary of the polyclinic administered VAS questionnaire to patients that reported flank pain. VAS scoring was carried out using a 0-1-2-3-4 scale. After the definitive diagnosis was made as a result of all evaluations, the patients were classified into two groups: renal colic group (group 1) and the LBP group (group 2). Patients with LBP were directed to the relevant clinics after being evaluated for renal colic. Later, they were contacted by phone and asked about their diagnosis and were included in group 2 if they fit the inclusion criteria. Data including age, gender, body mass index (BMI), type of pain, pain side, pain spread, location of pain, VAS score, presence of nausea-vomiting, fever or dysuria, costovertebral angle tenderness (CVAT), erythrocyte and leukocyte count in complete urinalysis, blood urea nitrogen (BUN), creatinine (Cre) level, degree of hydronephrosis on US or CT, presence of stone on US or CT, and stone size parameters were recorded. At the end of the third month, the number of patients that fit the inclusion criteria in group 1 reached 36, and in group 2 it reached 30. The power analysis with these patient numbers indicated that alpha was 0.05, while power was 87.6%. After the patient numbers were found to be sufficient, the follow-up was concluded and the analysis was initiated.

The patients who have one or more exclusion criteria were excluded prior to the study due to their history or just after the detection of the criteria. The pediatric age group was excluded from the study due to the presence of growing pains in this age group. Individuals aged ≥70 years were also excluded from the study because of common osteoporotic and chronic pain in the elderly. Thus, only participants aged 18-70 years were included in the study. Patients with lumbar disc herniation and pain due to neurological deficits were excluded. Wrestlers and athletes that have frequent muscle pain were also excluded. Patients with urinary anomalies that could cause hydronephrosis (ureter strictures, vesicoureteral reflux, etc.), trauma, history of malignancy, and gastrointestinal symptoms were also excluded from the study. Patients who showed systemic inflammatory symptoms with pain or who described recurrent inflammatory attacks or who had a diagnosis of a chronic inflammatory disease were also excluded from the study. In addition, patients with isolated urinary infections despite absence of stones in the examinations were also excluded. Since the aim of the study was to distinguish patients with LBP who frequently apply to the urology clinic from renal colic patients, only patients that applied to the urological outpatient clinic were included in the study. Patients that had previously applied to the emergency department or physical therapy outpatient clinic and were referred to urology were not included in the study.

The distribution of the groups was evaluated by Kolmogorov-Smirnov and Shapiro-Wilk tests. Representations of numerical data showing nonparametric distribution were given as median (min-max). Mann-Whitney U test was used for comparisons. Comparisons of categorical variables were made using the chi-square test. The patients’ definitive diagnoses obtained after completion of all evaluations were considered as dependent variables. The definitive diagnostic variables were described as renal colic and LBP. The VAS scores of these groups were considered as independent variables and binominal logistic regression analysis was applied. The receiver operating characteristic (ROC) curve analysis was used to identify a VAS score cutoff value in differentiation between renal colic and LBP. A p-value of <0.05 was considered significant.

## Results

Demographic and clinical data of the patients are given in table 1. The mean age of group 2 was significantly higher (p = 0.043). Pain lateralization was significantly more frequent on the right side (p = 0.008). Most of the patients in group 1 and none in group 2 had macroscopic hematuria (p = 0.029). In group 2, 62% of the patients did not have CVAT. In terms of CVAT presence, there was statistically significant difference between the groups (p < 0.0001). The mean VAS scores were also significantly higher in group 1 than in group 2 (p < 0.0001). When the laboratory findings of the patients were examined, all parameters, except pyuria, were significantly higher in group 1 (p > 0.05).

**Table 1:** Demographic and clinical data of the patients. Numerical values were given as median (min-max) and categorical values were given as n (%). BUN: blood urea nitrogen; BMI: body mass index; CVAT: costovertebral angle tenderness; US: ultrasonography; CT: computed tomography; VAS: visual analogue scale. **As there were empty slots, chi-square analysis could not be possible and p values could not be provided for these parameters; p < 0.05.

Parameters		Renal colic (n=36)	Lumbago (n=29)	p-value
Age		35 (21-69)	43 (20-71)	0.043
Gender	Female (30)	9 (30.0%)	21 (70.0%)	<0.0001
	Male (35)	27 (77.1%)	8 (22.9%)	
BMI		25.96 (21.05-29.05)	26.44 (19.10-35.49)	0.29
Pain type	Stabbing	16 (51.6%)	15 (48.4%)	0.73
	Dull	20 (58.8%)	14 (41.2%)	
Pain lateralization	Right	16 (55.2%)	13 (44.8%)	0.008
	Left	19 (70.4%)	8 (29.6%)	
	Bilateral	1 (11.1%)	8 (88.9%)	
Spread of pain	Present	15 (55.6%)	12 (44.4%)	1.0
	Absent	21 (55.3%)	17 (44.7%)	
Pain location**	Lumbar	21 (56.8%)	16 (43.2%)	
	Lumbar+ Inguinal	12 (66.7%)	6 (33.3%)	
	Back	0 (0%)	6 (100%)	
	Inguinal	1 (100%)	0 (0%)	
	Abdominal	2 (66.7%)	1 (33.3%)	
Macroscopic haematuria	Present	6 (100%)	0 (0%)	0.029
	Absent	30 (50.8%)	29 (49.2%)	
Nausea and/or vomiting	Present	8 (80%)	2 (20%)	0.16
	Absent	28 (50.9%)	27 (49.1%)	
Fever**	Present	1 (100%)	0 (0%)	
	Absent	35 (54.7%)	29 (45.3%)	
Dysuria	Present	11 (68.8%)	5 (31.3%)	0.34
	Absent	25 (51%)	24 (49%)	
CVAT	Absent	4 (18.2%)	18 (81.8%)	<0.0001
	Present	32 (74.4%)	11 (25.6%)	
BUN		29.5 (10-48)	21 (13-45)	0.009
Creatinine		0.85 (0.5-1.6)	0.7 (0.6-0.9)	0.002
VAS score		6 (1-10)	4 (2-5)	<0.0001
Hematuria		37 (0-1000)	2 (0-20)	<0.0001
Pyuria		2.5 (0-1000)	2 (0-19)	0.38

According to the results of logistic regression analysis, the possibility of renal colic increased 5.4 times more per one-unit increase in the VAS score compared to the possibility of lumbago (Table 2).

**Table 2:** Logistic regression analyses of VAS and renal colic VAS score is positively correlated with having renal colic. The likelihood of having renal colic increases 5.42-fold per 1-point increase in VAS score. VAS: visual analogue scale; SE: standard error.

B	SE	Odds ratio	p-value	95% CI
1.69	0.404	5.42	<0.0001	2.45-11.96

**Figure 1:**
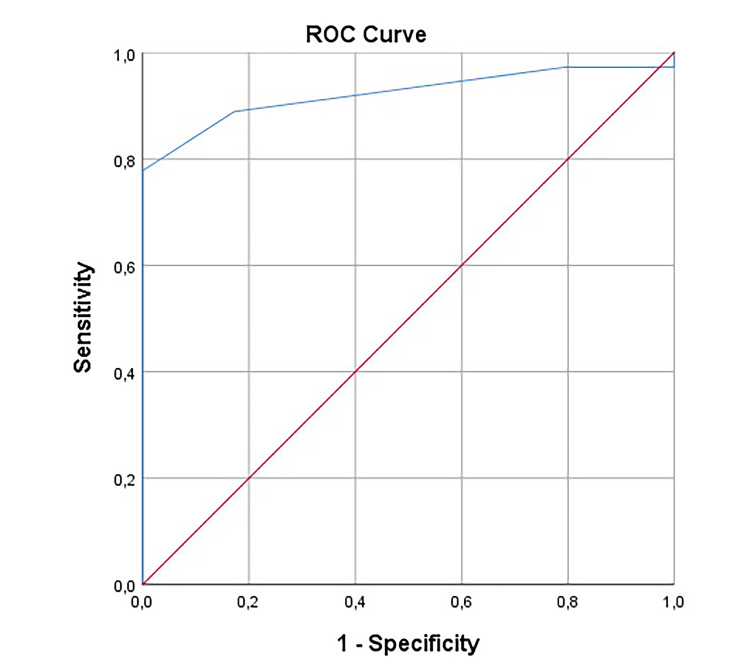
ROC curve of the VAS score in distinguishing lumbar back pain and renal colic. ROC: receiver operating characteristic; VAS: visual analogue scale.

In the ROC analysis, the area under the curve (AUC) was 0.92 (Figure 1, Table 3). Accordingly, there was a strong relationship between the VAS score and the distinction between renal colic and lumbago. When the cutoff values were examined, the highest sensitivity was 97% at the VAS score of 1.5 and the highest sensitivity was 78% when the VAS score was 5.5. However, in both measurements, the likelihood ratio was under 2. The highest likelihood ratio belonged to the VAS score value between 4.5 and 5.1. When the VAS was 4.5, the diagnosis of renal colic could be made with 88% sensitivity and 71% specificity.

**Table 3:** ROC analysis. ROC: receiver operating characteristic; AUC: area under curve; SE: standard error.

AUC	SE	95%CI	p-value	Cutoff	Sensitivity (%)	Specificity (%)
0.922	0.04	0.85-0.99	<0.0001	4.5	88.9	71.6

## Discussion

Renal colic due to urinary stones is commonly seen in both the urology clinic and the emergency department, and often has a similar manifestation with LBP [6]. Nowadays, diagnosis of renal colic can be made with high accuracy by using CT [5]. In evaluating such patients, clinicians encounter many factors such as the requirement of providing the most accurate diagnosis, meeting patients’ expectations, radiation exposure, and the procedures’ cost covered by insurance companies, especially in developed countries. However, considering the increased rates of CT performed due to the pre-diagnosis of renal colic in the last decades, it can be elaborated that physicians are more concerned about making an accurate diagnosis [7]. It has been determined that only 50% of the CT scans performed with the pre-diagnosis of renal colic detected stones and only 20% of those required further intervention [9].

Clinicians can make prediction of urinary stone disease based on the patient’s examination. However, since this prediction remains completely subjective, physicians frequently resort to some diagnostic tools such as CT. There are studies that state that the severity of pain in renal colic may lead to some physicians suspecting presence of renal stones [13]. In our interviews with both our patients and colleagues over the years, we also observed that pain severity is the most important physical examination factor in forming a subjective prediction for renal colic. Kuehhas et al. evaluated the VAS score in patients with renal colic and reported that the colic pain was extremely severe and the size of the stone correlated with the VAS score [14]. Şaşmaz et al. also stated that stone size and hydronephrosis increased pain intensity [15]. Many times clinicians make a pre-diagnose of urolithiasis regarding severity of pain [16]. Although we do not have a definitive answer on where the renal colic pain would rank if all pain in the body were scored since pain is individually subjective, there seems to be a consensus on the point that renal colic pain is indeed quite severe. In this study, the median value of VAS score was 6 for group 1 and 4 for group 2, which suggested that renal colic pain was more severe than LBP pain. In addition, the detection of renal colic was at a rate of 74.4% when CVAT was present on physical examination. Therefore, we believe that putting the concept of pain severity into the center of stone prediction is a logical approach.

A large group of diseases including inflammatory diseases associated with the musculoskeletal system, malignancies, pregnancy, trauma, osteoporosis, nerve root compression, radiculopathy, plexopathy, degenerative disc disease, disc hernia, spinal stenosis, sacroiliac joint dysfunction, facet joint damage, infection are suspected in the differential diagnosis of renal colic [17]. These pains are grouped under the name of LBP and first of all, the distinction between mechanical pains and radiculopathies are made. Mechanical pains (pains related with environmental factors and physical activity) are the most common pains. In this group, lumbago (spasm in quadratus lumborum muscle or paraspinal muscles), injuries in spines, intervertebral discs and their surrounding soft tissues, disc herniation, spondylolisthesis and pregnancy are common causes of pain. Medical history and physical examination can often be used to recognize degenerative, oncological, and infectious pain. Inflammatory pain is often associated with systemic symptoms that require laboratory analysis and further assessment beyond standard evaluation. Therefore, the type of pain that can be confused with renal colic most often is mechanical low back pain. Mechanical low back pain, especially the types of pain that give neurological symptoms (disc herniation, fractures, etc.), can be defined with symptoms such as limitation of movement, paresis, and plegia. Thus, lumbago-like soft tissue-related pain seems to be more likely to be confused with renal colic, and it can be argued that they are in the potential suspect disease group for unnecessary CT scans [17]. In this study, we learned that all 30 patients that were referred to other clinics after urological examination were diagnosed with mechanical low back pain.

It is clear that urologists and emergency physicians need simple predictive tools to decide whether or not to perform a CT scan. Researchers have created various scoring systems to increase stone prediction. In 2014, Moore et al. developed the STONE (Sex, Timing, Origin, Nausea, Erythrocytes) score [9]. They reported the risk of stone detection as 8% in the low-risk group, 51% in the medium-risk group, and 89% in the high-risk group. Rapp et al. developed Vstone in 2016 [12]. Their evaluation parameters included flank pain, hematuria, nausea vomiting, and a history of stone disease. Al-Kadhi et al. validated the fast track renal colic (FTRC) tool created by NICE (National Institute for Health and Care Excellence) on 1,157 patients [11,18]. The purpose of FTRC was to assist nurses when triaging patients with suspected renal colic by referring patients with a single kidney, dehydration, pre-shock or shock symptoms directly to the emergency physician, while directing other patients to analgesia and then to the urologist. If the patients are suitable for the criteria, renal colic can be considered via fast track and patients could be provided with rapid analgesia and their consult with the urologist could be accelerated. In 2017, Fukuhara et al. developed CHOKAAI (distension of kidney Capsule, Hydronephrosis, Occult blood in urine, Kidney stone history, Adult, Age, Diminution of pain within six hours) scoring system [10]. In 2018, Başeskioğlu et al. designed the Osmangazi University Score, which used nausea, a history of stones, Cre, and hematuria [5]. In a meta-analysis that included the aforementioned scoring systems along with six others, Mirfazaelian et al. found the STONE scoring system to be superior to others [1,19-22]. The same year, Fukuhara et al. reported that the CHOKAAI score was superior to STONE [23].

The common parameter used in all aforementioned studies was the presence of hematuria. In addition, data on nausea-vomiting symptoms, presence of stone history and timing of pain were frequently used in studies. In our study, we also evaluated these parameters. Hematuria, a parameter that can be considered as stone-specific, shows a very high prevalence in patients with renal colic [24]. In our study, although there was a significant difference between groups in paired comparisons, we observed that 10 patients with LBP had microscopic hematuria without significant findings on CT or US, and this hematuria disappeared after symptomatic treatment. In their meta-analysis, Minotti et al. also emphasized that microhematuria was not sufficient to be a stone-specific symptom [24]. Again, it is remarkable that nausea-vomiting, which is thought to develop secondary to tension in the kidney capsule and hydronephrosis, was not observed in most of our patients, but was seen in a small number of patients with LBP. However, there was no significant difference between the groups. Parameters such as dysuria, the extent of pain, and the nature of the pain that are known to be classical manifestation of stone were also not significantly different between the groups. It is quite possible for the clinical findings of these two patient groups to be confused with each other because of bladder symptoms or pain locations could be misleading [13,25].

Hematuria, VAS score, BUN, Cre levels, age, gender, lateralization of pain, and CVAT presence showed significant differences between the groups in pairwise comparison. All of these parameters, except the VAS score, lost their significance in logistic regression. In the comparison between the groups, the VAS score was much higher in patients with renal colic. The possibility of renal colic increased by 5.4 times with every 1-unit increase in the VAS score. The cutoff value for VAS score was 4.5. Considering this cutoff value, while the VAS score of 4 was the upper limit for LBP, the VAS score of 5 was the lower limit for renal colic. In other words, it would not be wrong to say that we documented the examination-based prediction with a numerical scale.

We retrospectively examined how our VAS score-based renal stone prediction model worked in our study. We found that surgeons evaluated 22 patients in group 2 by US. Only three of these patients had a VAS score of 5, while the rest had a score of 4 or less. There were two patients that underwent CT after US, one of them had a VAS score of 2, while the other had a VAS score of 4. The number of patients for whom our surgeon directly requested CT without ultrasound was 7. One of these patients had a VAS score of 5 and the rest of the patients had a VAS score of ≤4. If the VAS score-based model had been used here, there would have been only 3 US examinations, instead of 22 (86.3% reduction) and 1 CT examination instead of 9 (88.8% reduction) and patients would have been saved from unnecessary examination and time loss. We can even suggest that this prediction model can save patients from unnecessary laboratory tests. All of these patients had normal Cre levels and only 4 patients had microscopic hematuria on complete urinalysis. The highest number of erythrocytes in patients that underwent CT was reported as 11. It can be thought that a false-positive urinalysis could be misleading for the clinician in terms of CT usage. In group 1, among the patients with renal colic, the VAS score was 1 in only one patient, and was 4 in three patients. Unfortunately, three of these four patients underwent a CT scan and all of them passed stones with only medical expulsive therapy.

The evaluation of VAS score may also have other benefits in the renal stone treatment course. Papa et al. reported that the VAS score could also be used as a predictive tool for whether or not patients will need intervention in the period after their first admission [2]. According to this, they emphasized that the rates of referral to urology would decrease from 88% to 49% when the patients that applied to the emergency department and received pain palliation were evaluated based on the size of the stone being 6 mm and above, the location of the stone on the midureter and the VAS score for residual pain being 2 and above. Since our study has already been conducted in the urology clinic, we think that studies covering both before and after evaluation will provide more comprehensive information about the use of the VAS score in terms of intervention prediction.

Considering the cost of CT imaging and the problem of radiation exposure, we think that this study has achieved a useful result in both reducing the cost and protecting the patients from radiation risk by offering a new approach to distinguish between patients with LBP and renal stones. We also believe that it offers an easy-to-apply exit door to overcome the conflict between the stress of making an accurate diagnosis and ethical and social issues such as cost and radiation burden. Since we did not do cost analysis and such a questionnaire was not applied to physicians in our study, we think that these additional benefits could be revealed with future studies. In addition, the aim of our study was not to provide a scoring system, but to evaluate the use of VAS scoring in the differential diagnosis of renal colic. Although we found the VAS score to be significantly sufficient with this pilot study, the false positivity and negativity rates to be obtained with prospective randomized studies will help with validation of this approach. We believe that even if the VAS score alone is not considered sufficient, it should be included in the scoring systems.

Our study is the first study in the literature that shows that VAS scoring can be useful in distinguishing patients with LBP who frequently apply to the urology outpatient clinic from patients with acute renal colic. The very high AUC value of 92% that we obtained in this study is an encouraging finding and could be a basis for other studies. Since the power analysis of our study was 87.6%, the number of patients in each group was considered sufficient. Despite this, our study has some limitations such as retrospective design, small number of our patients with VAS scores of 1, 2 and 10, the narrow spectrum of LBP subdiagnoses in group 2 due to the limited follow-up, possibility of regional variation of the data due to single-center design, and lack of data regarding pain onset and duration. The patient count would easily be raised by conducting a study with having a support from emergency or physical medicine and rehabilitation clinics. More studies involving these departments will provide more patient counts and verify the results of this study. We only used a 0-1-2-3-4 scale for evaluating VAS in this study. The ease of VAS use might differ among populations and other VAS tools may benefit. Although this study was not conducted to compare the VAS tools in making differential diagnosis between LBP and renal colic, this might be considered as a future aspect of this study. Also, the strength of foreseeing based on VAS scoring should be tested in future studies which involve other LBP types that were excluded from this study. Multi-center prospective randomized studies will provide more clear information about the place of VAS in the differential diagnosis of LBP and renal colic.

## Conclusions

The findings of this study revealed that stone pains are more severe than LBP without movement limitation. If the VAS score is ≤ 4 in patients that have flank pain without limitation of movement at the time of admission to the urology outpatient clinic, the treatment of these patients can be managed with a simple medical treatment plan or priority can be given to referral to relevant polyclinics without performing basic examinations such as complete urinalyses, Cre, US or advanced examinations such as CT. In these patients, unnecessary US scans be reduced by 86.3% and unnecessary CT scans by 88.8%. A VAS score of ≥5 should warn the clinician about the necessity of routine urinary stone examinations. In this study, all patients with stones whose VAS score was ≤ 4 passed their stones with simple medical treatment and had no complications. Consequently, the use of the VAS score in the initial evaluation of patients with mechanical LBP and renal colic in the outpatient room can lead the clinician to the correct diagnosis without the need for basic and/or advanced examination.
